# Paramedic Out-of-hospital Cardiac Arrest Case Volume Is a Predictor of Return of Spontaneous Circulation

**DOI:** 10.5811/westjem.2018.3.37051

**Published:** 2018-05-15

**Authors:** Jenna E. Tuttle, Michael W. Hubble

**Affiliations:** Western Carolina University, School of Health Sciences, Emergency Medical Care Program, Cullowhee, North Carolina

## Abstract

**Introduction:**

Many factors contribute to the survival of out-of-hospital cardiac arrest (OHCA). One such factor is the quality of resuscitation efforts, which in turn may be a function of OHCA case volume. However, few studies have investigated the OHCA case volume-survival relationship. Consequently, we sought to develop a model describing the likelihood of return of spontaneous circulation (ROSC) as a function of paramedic cumulative OHCA experience.

**Methods:**

We conducted a statewide retrospective study of cardiac arrest using the North Carolina Prehospital Care Reporting System. Adult patients suffering a witnessed, non-traumatic cardiac arrest between January 2012 and June 2014 were included. Using logistic regression, we calculated an adjusted odds ratio (OR) for the influence of the preceding five-year paramedic OHCA case volume on ROSC while controlling for the potentially confounding variables identified a priori as patient age, gender, and non-Caucasian race; shockable presenting rhythm; layperson/first responder cardiopulmonary resuscitation (CPR); and emergency medical services (EMS) response time.

**Results:**

Of the 6,405 patients meeting inclusion criteria, 3,155 (49.3%) experienced ROSC. ROSC was more likely among patients treated by paramedics with ≥ 15 OHCA experiences during the preceding five years (OR [1.21], p<0.01). ROSC was also more likely among patients with shockable initial rhythms (OR [2.35], p<0.01) and who received layperson/first responder CPR (OR [1.77], p<0.01). Increasing patient age (OR [0.996], p=0.02), male gender (OR [0.742], p<0.01), and increasing EMS response time (OR [0.954], p<0.01) were associated with a decreased likelihood of ROSC. Non-Caucasian race was not an independent predictor of ROSC.

**Conclusion:**

We found that a paramedic five-year OHCA case volume of ≥ 15 is significantly associated with ROSC. Further study is needed to determine the specific actions of these more experienced paramedics who are responsible for the increased likelihood of ROSC, as well as the influence of case volume on the longer-term outcome measures of hospital discharge and neurological function.

## INTRODUCTION

Sudden cardiac death accounts for more than half of all coronary heart disease deaths in the United States (U.S.), with approximately 326,200 cases of out-of-hospital cardiac arrest (OHCA) patients assessed by emergency medical services (EMS) each year.^1^ The importance of bystander cardiopulmonary resuscitation (CPR), early defibrillation, and quality resuscitation and post-resuscitation care on favorable outcomes are well documented. However, other factors such as the quality and timing of paramedic interventions may also influence outcomes. Unfortunately, resuscitation skills are known to decline over time,^2^ which may lower survival rates. Such skill decay may be the result of limited exposure to OHCA case volume, which has been observed to average less than two cases per year per paramedic in some areas.^3^ Only one study has previously quantified the OHCA case volume-survival relationship among paramedics;^4^ however, it is unclear if the findings of this international study can be extrapolated to EMS systems in the U.S.

Due to the lack of previous investigations among U.S. EMS systems, the influence of OHCA case volume on patient outcomes remains poorly quantified. Therefore, using a statewide dataset we sought to develop a model describing the likelihood of return of spontaneous circulation (ROSC) as a function of OHCA case volume. We hypothesized that the likelihood of ROSC increased with increasing paramedic OHCA case volume.

## METHODS

### Data Sources

With institutional review board approval from Western Carolina University, we conducted a retrospective observational study of the influence of cardiac-arrest case volume on ROSC using the North Carolina Prehospital Care Reporting System (PreMIS) database. PreMIS is the data collection and management system that collects statewide data from over 400 North Carolina EMS agencies. Data are submitted to PreMIS for all EMS responses in North Carolina, and the data points for collection are a subset of the National Emergency Medical Services Information System (NEMSIS) dataset.^5^

### Outcome Measures

The primary outcome measure was prehospital ROSC. We did not make any distinction between transient or persistent ROSC.

### Study Setting

North Carolina is the nation’s ninth most populous state with approximately 10 million people dispersed across a land mass of 48,617 square miles.^6^ Demographically, the state is comprised of urban, suburban, and rural populations, with 33.9% of the population living in rural areas.^7^ Cardiovascular disease is the second leading cause of death in the state, resulting in 18,467 deaths in 2015.^8^

### Sample

We queried the PreMIS database to identify individuals who suffered a cardiac arrest in North Carolina between January 1, 2012, and June 30, 2014. These records were then filtered to meet our inclusion and exclusion criteria. Inclusion criteria consisted of all adult patients (≥18 years) suffering a bystander- or EMS-witnessed, non-traumatic cardiac arrest. The PreMIS database was then queried by the primary paramedic attending to each patient in the sample to determine his/her number of cardiac arrest cases treated in the previous five years. In determining the historical OHCA case volume, no distinction was made as to whether the paramedic was the primary attending paramedic, or “code leader,” or assumed a secondary (“skills”) role on the resuscitation team. We believed that any experience in OHCA resuscitation, whether in a primary or secondary role, would contribute positively to the cumulative resuscitation experience.

**Population Health Research Capsule**

What do we already know about this issue?

Many factors contribute to the survival of out-of-hospital cardiac arrest (OHCA). One such factor is the quality of resuscitation efforts, which in turn may be a function of OHCA case volume.

What was the research question?

To quantify the OHCA case volume-survival relationship.

What was the major finding of the study?

Paramedic five-year OHCA case volume of ≥ 15 is significantly associated with return of spontaneous circulation.

How does this improve population health?

Strategies to increase paramedic cardiac arrest case volume or exposure to high fidelity simulation have the potential to prevent resuscitation skill decay and improve OHCA survival.

### Statistical Analysis

We analyzed abstracted data using IBM SPSS Statistics version 24 (IBM Corporation, Somers, NY) with p ≤ 0.05 indicating statistical significance. Continuous variables and time intervals are presented as means (standard deviation), and categorical variables are presented using frequency distributions and percentages. We compared continuous variables using Student’s t-test or the Mann-Whitney test. Categorical data were analyzed using the chi square test, continuity correction, or Fisher’s exact test as appropriate. We calculated an adjusted odds ratio (OR) for the influence of OHCA case volume using logistic regression to control for potentially confounding variables identified a priori as patient age, gender, and non-Caucasian race; shockable presenting rhythm; layperson/first responder CPR; and EMS response time.

## RESULTS

During the study period, 8,790 patients met inclusionary criteria. Of these, 2,385 were excluded due to incomplete data elements. Of the 6,405 patients included in the analysis, the mean age was 66.5 (±15.2) years and males accounted for 61.7% of the sample. A shockable rhythm was the first presenting rhythm upon EMS arrival in 30.0% of cases. The mean EMS response time, measured as call receipt to scene arrival, was 8.3 (±4.8) minutes, and layperson/first responder CPR was performed prior to EMS arrival in 44.0% of cases. In total, 3,155 patients (49.3%) experienced ROSC. The lead paramedics attending the patients in the database had participated in an average of 23.6 (±20.3) OHCA cases in the previous five years, either as the “code leader” or in a secondary role. Additional details of the sample are provided in Table 1.

The results of the univariate analysis of ROSC are presented in Table 2. Notably, compared to patients without ROSC, a greater proportion of patients with ROSC received layperson/first responder CPR (60.0% vs. 51.4%, p = 0.03) and presented with a shockable rhythm (38.9 vs. 21.2%, p < 0.01), but were less likely to be male (60.1% vs. 63.2%, p < 0.01). Patients with ROSC also had shorter EMS response times (7.7 vs. 8.9 minutes, p < 0.01) and were treated by paramedics with greater five-year cumulative OHCA experience (24.5 vs. 22.7, p<0.01).

We used logistic regression to control for potentially confounding variables. Based on clinical reasoning, the following variables were entered into the model: paramedic OHCA experience ≥15 in the previous five years; patient age, gender, and non-Caucasian race; shockable presenting rhythm; layperson/first responder CPR; and EMS response time. OHCA case volume was defined as a binary variable of participation in ≥ 15 previous resuscitation attempts. This level of case volume was selected because the probability of ROSC by OHCA case volume appeared to plateau around 15 previous arrests (Figure).

ROSC was more likely when the patient was treated by a lead paramedic who had attended 15 or more cardiac arrests in the previous year (OR [1.21], p<0.01), and less likely with increasing age (OR [0.99], p <0.02) and EMS response time (OR [0.95], p<0.01). Compared to patients with non-shockable rhythms, patients with shockable rhythms were more likely to achieve ROSC (OR [2.35], p<0.01). ROSC was more likely among patients receiving layperson/first responder CPR (OR [1.77], p<0.01) and less likely among males (OR [0.74], p<0.01). Non-Caucasian race was not an independent predictor of ROSC. Details on the logistic regression results for ROSC are provided in Table 3.

With the exception of scene arrival to administration of the first vasopressor time interval, there were no differences in the time required to perform on-scene skills between paramedics with and without 15 or more cumulative OHCA experiences (Table 4).

## DISCUSSION

This study is the first to examine the relationship between paramedic OHCA case volume and ROSC in a U.S. EMS system. We found that patients treated by paramedics with 15 or more OHCA exposures in the previous five years were 21% more likely to attain ROSC. Few previous studies have investigated this relationship among paramedics, and none have done so in a U.S. EMS system.

Dyson et al. measured the association between paramedic OHCA exposure and patient survival in Victoria, Australia.^4^ In their study they found that OHCA exposure during the preceding three years had a positive impact on patient survival. The odds of survival increased for every additional increase in the median OCHA exposure. Compared with patients treated by paramedics with a median of ≤ 6 arrests during the preceding three years, the odds of survival were higher for patients treated by paramedics with 7–11 (OR [1.26]), 12–17 (OR [1.29]), and >17 (OR [1.50]) OHCA exposures. Interestingly, they did not find any relationship between paramedic career experience and survival, suggesting that career longevity alone does not convey any benefit in terms of patient outcomes following OHCA.

Another salient finding by Dyson et al. was that patient survival decreased when six months or more had lapsed since the previous OHCA exposure.^4^ They noted that this time frame is similar to the post-training decay rate of advanced life support skills after training, reported by Yang et al.^2^,^4^

The only previous investigation involving a U.S. EMS system was conducted in King County, Washington, by Gold and Eisenberg.^9^ Although they did not specifically evaluate the impact of OHCA exposures on survival, they did examine the influence of the number of years of paramedic career experience of the primary (code leader) and secondary (skills) paramedic on patient survival.^9^ They found no association between years of paramedic experience and survival for the primary paramedic (OR [1.01], 95% confidence interval [CI] [0.99–1.03]), but they did find a positive relationship between experience and survival for the secondary paramedic (OR [1.02], 95% CI [1.00–1.04]). They speculated that treatment of cardiac arrests tends to be protocol-driven events and on-scene decision-making, and ultimately survival, is not sensitive to paramedic career experience. In contrast, they surmised that the “skills paramedic” did become more proficient at rendering treatments as career experience increased and this resulted in improved outcomes. However, they did not report skills success rates or time to treatments, so it is unclear if these measures were actually influenced by career experience. In our dataset, we did not find any improvement in on-scene performance between lead paramedics with and without 15 or more OHCA experiences other than a shorter time to administer the first dose of vasopressors for the more experienced paramedics (Table 4). Unfortunately, we did not have data to compare on-scene performance of the secondary (skills) paramedics with respect to cumulative OHCA experience.

Suspecting a link between endotracheal intubation (ETI) experience and OHCA survival, Wang et al. compared outcomes among paramedics with low (1–10 tracheal intubations in the preceding six years), medium (11–25 tracheal intubations), high (26–50 tracheal intubations), and very high (greater than 50 tracheal intubations).^10^ After adjusting for factors known to influence patient outcome and using low cumulative experience as the reference category, they found a significant survival benefit among paramedics with very high ETI exposure (OR [1.48], 95% CI [1.15–1.89]).^10^ This finding lends credence to the hypothesis of Gold and Eisenberg that OHCA survival is influenced by increased levels of proficiency of the “skills paramedic” rather than the team leader whose decision-making role is somewhat dictated by protocol.^9^ In our study we focused only on OHCA exposure and did not evaluate cumulative skills experience.

If our findings and those of Dyson et al. are accurate, then a case volume-survival relationship exists between paramedics’ OHCA experience and patient survival.^4^ In general terms, this relationship is not unique to EMS and OHCA as clinical case volume has been linked to patient outcomes in other settings and patient conditions.^11^–^13^ The greater issue, then, is devising strategies to ensure that paramedics have adequate case volumes to obtain and maintain proficiency in OHCA management. In the study by Dyson et al. on average, paramedics were exposed to two OHCA per year and 10% of their workforce had no OHCA exposure during their seven-year study.^4^ In our study, patients were treated by paramedics who averaged 24 OHCA during the preceding five years, yet 41% of the patients were treated by paramedics who had fewer than the 15-case threshold that was associated with increased odds of ROSC. Combined, these studies suggest that other forms of skills maintenance are needed.

To address the problem of infrequent exposure to specific patient populations and skills opportunities, some EMS systems have instituted strategies whereby paramedics specialize in certain patients or procedures, such as cardiac arrest.^14^ These paramedics are then dispatched to all relevant calls in an effort to coalesce experience among a smaller group of clinicians. Such strategies mimic the rapid response team approach used in hospitals, which has been demonstrated to reduce mortality.^15^–^17^ The disadvantage to this strategy is that turnover may eventually exhaust this cadre of highly experienced clinicians that must then be replaced with clinicians who have had relatively few cumulative exposures. Thus, this may only be a short-term strategy that ultimately results in minimal experience among the bulk of clinicians. Moreover, we were unable to identify any published reports on the effectiveness of this strategy.

Another approach to maintaining skills proficiency is through high-fidelity simulation. This strategy has been used successfully to increase survival rates in the hospital setting.^18^,^19^ However, its use in EMS training programs varies,^20^ and there are no published reports correlating simulation training among paramedics with improved OHCA outcomes. Nonetheless, in low call-volume settings this may be the best option to maintain resuscitation skills.

## LIMITATIONS

Our study is subject to the usual limitations of a retrospective design, including the accuracy of data collection and the potential for measured and unmeasured confounders that may account for the observed outcomes. In addition, our study focused exclusively on the influence of the “code leader.” Consequently, we did not evaluate the influence of the “skills paramedic” on skills success rates or ROSC. Although we found a relationship between OHCA case volume and ROSC, we do not know the reason for these differences. Additional study is needed to investigate the specific resuscitation traits of the more experienced paramedics that might explain their increased likelihood of attaining ROSC. Moreover, additional studies are needed to correlate these findings with longer-term outcomes such as hospital discharge and neurological function.

## CONCLUSION

Within the limits of our study design, we found that a paramedic five-year OHCA case volume of ≥ 15 is significantly associated with ROSC. Further study is needed to determine the specific actions of the more experienced paramedics that are responsible for the increased likelihood of ROSC, as well as the influence of case volume on the longer-term outcome measures of hospital discharge and neurological function.

## Figures and Tables

**Figure:**
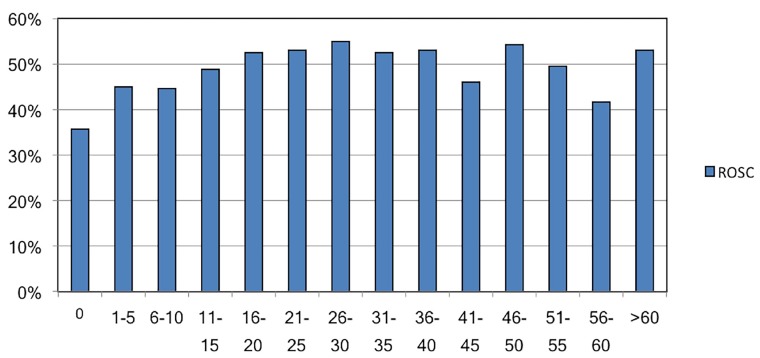
Percentage return of spontaneous circulation (ROSC) by paramedic cumulative out-of-hospital cardiac arrest (OHCA) cumulative case volume. Proportion of patients attaining ROSC by paramedic five-year OHCA case volume.

**Table 1:** Demographics of patients enrolled in a study of the effect of paramedic out-of-hospital cardiac arrest (OHCA) case volume on return of spontaneous circulation (ROSC).

Characteristic	Result
Paramedic OHCA experience over previous 5 years (mean, SD)	23.6 (±20.3)
Male (%)	61.7
Non-Caucasian (%)	30.8
Age (mean, SD)	66.5 (±15.2)
Shockable rhythm (%)	30.0
Layperson/first responder CPR (%)	44.0
EMS response time in minutes (mean, SD)	8.3 (±4.8)
ROSC (%)	49.3

*SD*, standard deviation; *CPR,* cardiopulmonary resuscitation; *EMS*, emergency medical services.

**Table 2:** Univariate comparison of patients enrolled in a study to determine the effect of paramedic OHCA case volume on ROSC comparing patients with and without ROSC.

Variable	ROSC	No ROSC	P value
Paramedics with prior OHCA case volume ≥ 15 (%)	24.5	22.7	<0.01
Male gender (%)	60.1	63.2	0.01
Non-Caucasian (%)	31.7	29.8	0.09
Age in years (mean, ± SD)	65.7	67.4	<0.01
Shockable rhythm (%)	38.9	21.2	<0.01
Layperson/first responder CPR (%)	51.8	36.3	<0.01
EMS response time in minutes (mean, ± SD)	7.7	8.9	<0.01

*OHCA*, out-of-hospital cardiac arrest; *ROSC*, return of spontaneous circulation; *SD*, standard deviation; *CPR*, cardiopulmonary resuscitation; *EMS*, emergency medical services.

**Table 3:** Adjusted odds ratios for selected predictors of return of spontaneous circulation (ROSC) among patients enrolled in a study of the effect paramedic out-of-hospital cardiac arrest (OHCA) case volume on return of spontaneous circulation.

	ROSC		
	
Variable	Adjusted odds ratio	P value	95% CI
Prior OHCA case volume ≥ 15	1.217	<0.01	1.109–1.355
Male gender	0.742	<0.01	0.667–0.827
Non-Caucasian	1.073	0.22	0.959–1.201
Age	0.996	0.02	0.993–0.999
Shockable rhythm	2.354	<0.01	2.096–2.644
Layperson/first responder CPR	1.773	<0.01	1.597–1.969
EMS response time in minutes	0.954	<0.01	0.943–0.964

*CPR*, cardiopulmonary resuscitation; *EMS*, emergency medical services.

**Table 4:** Comparison of scene events by paramedic 5-year out-of-hospital cardiac arrest (OHCA) case volume experience.

Scene events	<15 Cumulative OHCA resuscitations	≥15 Cumulative OHCA resuscitations	P value
Scene arrival to first defibrillation (minutes)^1^	7.25	6.80	0.36
Scene arrival to first advanced airway (minutes)	10.05	10.21	0.51
Scene arrival to first vasopressor administration (minutes)	10.04	9.33	<0.01
Scene arrival to first ROSC (minutes)	22.26	21.35	0.16

1 For patients presenting with a shockable rhythm upon EMS arrival.

*ROSC*, return of spontaneous circulation.
